# Premature ventricular contraction increases the risk of heart failure and ventricular tachyarrhythmia

**DOI:** 10.1038/s41598-021-92088-0

**Published:** 2021-06-16

**Authors:** Yun Gi Kim, Yun Young Choi, Kyung-Do Han, Kyoung Jin Min, Ha Young Choi, Jaemin Shim, Jong-Il Choi, Young-Hoon Kim

**Affiliations:** 1 Division of Cardiology, Department of Internal Medicine, Korea University Anam Hospital, Korea University College of Medicine, 73 Goryeodae-ro, Seongbuk-gu, Seoul, 02841 Republic of Korea; 2 Department of Statistics and Actuarial Science, Soongsil University, Seoul, Republic of Korea

**Keywords:** Cardiology, Cardiovascular biology

## Abstract

Premature ventricular contraction (PVC), a common arrhythmia affecting 1–2% of the general population, has been considered to have a benign clinical course. However, people with PVC often develop heart failure and ventricular arrhythmias such as ventricular tachycardia. We aimed to clarify the risk of heart failure and lethal ventricular arrhythmias in people with PVC. The Korean National Health Insurance Service database was used for this study. People who underwent nationwide health check-ups in 2009 were enrolled in this study and clinical follow-up data until December 2018 were analyzed. Newly diagnosed PVC in 2009 (≥ 1 inpatient or outpatient claim) were identified and cumulative incidence of heart failure (≥ 1 inpatient claim) and ventricular arrhythmias (≥ 1 inpatient or outpatient claim) were compared. A total of 4515 people were first diagnosed with PVC in 2009 among 9,743,582 people without prior history of PVC, heart failure, or ventricular arrhythmias. People with newly diagnosed PVC in 2009 had a significantly higher incidence of heart failure compared to those without PVC [adjusted hazard ratio (HR)  1.371; 95% confidence interval (CI)  1.177–1.598; *p * < 0.001]. Significant interaction was observed between age and PVC with young age people at greater risk of developing heart failure for having PVC. The incidence of ventricular arrhythmia was also significantly increased in people with PVC (HR  5.588; 95% CI  4.553–6.859; *p * < 0.001). Age and chronic kidney disease had significant interactions with PVC. In conclusion, the incidence of heart failure and ventricular arrhythmia was significantly increased in people with PVC. Outpatient follow-up of people with PVC can be helpful to detect early signs of heart failure or advanced forms of ventricular arrhythmia.

## Introduction

A significant proportion of the general population is affected by premature ventricular contraction (PVC)^1^. People with PVC suffer from associated symptoms such as palpitations, skipped beats, chest pain, and dizziness^2–4^. Although the adverse effects of PVC are usually limited to symptoms and impaired quality of life, it can provoke other medical conditions like PVC-induced heart failure, ventricular tachycardia, or ventricular fibrillation^2,4–6^. Our previous work suggested that PVC can also be associated with increased risk of atrial fibrillation and ischemic stroke. Therefore, PVC can be associated with major adverse cardiac events in a subset of patients.

The currently available evidence for PVC-induced heart failure is limited. Duffee and colleagues reported a significant improvement in left ventricular function after suppressing PVC with amiodarone in five patients^7^. Yarlagadda et al. also reported significant improvement in left ventricular systolic function after successful catheter ablation in patients with PVC^8^. However, evidence for chronological association between PVC and heart failure is currently lacking. The subsets of ventricular tachycardia (VT) and ventricular fibrillation (VF) are triggered by PVC and can be successfully ablated by targeting those triggering PVC^9,10^. However, the risk of developing VT or VF in patients with PVC remains to be elucidated, especially by chronological data and not just cross-sectional data. We aimed to evaluate the incidence of heart failure and lethal ventricular arrhythmias (VT, ventricular flutter, and VF) in people with and without PVC using the Korean National Health Insurance Service (K-NHIS) database.

## Methods

### Patients

We used the K-NHIS database to conduct this study. The majority of residents of the Republic of Korea are mandatory subscribers of K-NHIS, the single medical insurer managed by the government. Therefore, data obtained from the K-NHIS represents the entire population of the Republic of Korea. Healthcare information such as inpatient and outpatient service records; diagnostic codes for various disease such as PVC, heart failure, VT, ventricular flutter, and VF; prescription records; and mortality data are stored in the K-NHIS database. A nationwide health check-up service is provided regularly for all subscribers. People who underwent a nationwide health check-up at certain times can comprise a valuable research cohort with a significantly large sample size. If the study protocols are approved by the official review committee (https://nhiss.nhis.or.kr/), medical researchers are able to analyze healthcare information data stored in the K-NHIS database.

We analyzed people who underwent nationwide health check-ups in 2009. Medical data obtained from January 2002 to December 2008 were used as screening data to identify underlying medical history. Patients who had medical records of PVC, AF, ischemic stroke, heart failure, VT, or VF during the screening period were excluded from the analysis. People younger than 20 years were also excluded. Those who developed PVC in 2009 were defined as the study group and those who did not develop PVC as the control group. Clinical follow-up data were available until December 2018. Impact of PVC on heart failure and ventricular arrhythmia was evaluated. The institutional review board of Korea University Medicine Anam Hospital approved this specific study. Written informed consent was waived by the institutional review board of Korea University Medicine Anam Hospital due to the retrospective nature of this study. The ethical guidelines of the 2008 declaration of Helsinki and legal regulations of Republic of Korea were strictly adhered.

### Primary outcome endpoint

The primary outcome endpoint was the occurrence of heart failure and ventricular arrhythmia composite, which consisted of VT, ventricular flutter, and VF. People who underwent nationwide health check-ups in 2009 were divided into two groups: (1) those diagnosed with PVC in 2009 and (2) those not diagnosed with PVC in 2009. Follow-up data were available until 2018. The incidence of heart failure and ventricular arrhythmia composite was compared between people with and without PVC. Newly developed PVCs after 2009 was censored. The incidence of heart failure and ventricular arrhythmia composite was defined as the number of events calculated for 1000 person * years of follow-up.

### Definitions

Two criteria were used to identify people with PVC: (1) one outpatient record (PVC 1) or (2) two or more outpatient records or one inpatient record (PVC 2) of International Classification of Disease, Tenth Revision (ICD-10) codes in the K-NHIS database. People with highly symptomatic PVC will likely seek frequent medical assistance. Therefore, PVC 2 which was diagnosed with more intensified criteria can represent more symptomatic form of PVC.

Heart failure was diagnosed by one inpatient record for heart failure. Diagnosis of VT, ventricular flutter, and VF required one outpatient or inpatient record. The robustness of this definition has been validated in previous studies^11,12^. Diagnostic codes to identify PVC, heart failure, VT, ventricular flutter, VF, and SCD are described in Supplementary Table S1.

Data regarding age, sex, body mass index (BMI), smoking status, alcohol consumption status, and medical conditions such as hypertension, diabetes mellitus, chronic kidney disease, and dyslipidemia were retrieved from the K-NHIS database.

### Statistical analysis

Student’s *t* test was used for continuous variables and categorical variables were analyzed with chi-square test or Fisher’s exact test as appropriate. Kaplan–Meier curve analysis with log-rank *t* test was used to compare the cumulative incidence of heart failure and ventricular arrhythmia composite in people with and without PVC. Non-adjusted and adjusted hazard ratios (HR) with 95% confidence intervals (CI) were calculated with Cox regression analysis. Three different multivariate models were used: (1) model 1: adjusted for age and sex; (2) model 2: adjusted for age, sex, smoking status, alcohol consumption status, regular physical activity, and BMI; and (3) model 3: adjusted for age, sex, smoking status, alcohol consumption status, regular physical activity, BMI, hypertension, diabetes mellitus, and dyslipidemia. All tests were two-tailed, and *p * values ≤ 0.05 were considered statistically significant. All statistical analyses were performed with SAS version 9.2 (SAS Institute, Cary, NC, USA).

## Results

### Patients

**Figure 1:**
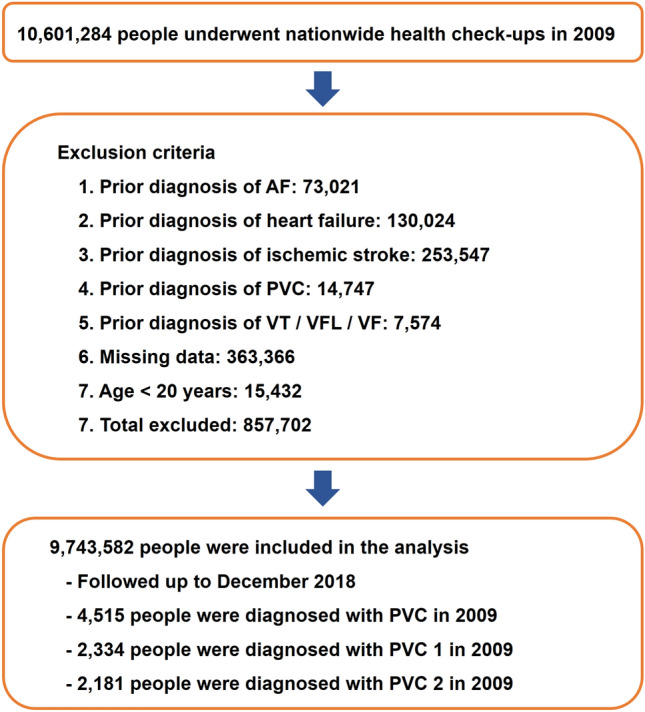
Flow diagram of the study. *AF* atrial fibrillation; *PVC* premature ventricular contraction; *SCD* sudden cardiac death; *VF* ventricular fibrillation; *VFL* ventricular flutter; *VT* ventricular tachycardia.

In 2009, 10,601,284 people underwent nationwide health check-ups. People who were (1) under 20 years or (2) had a prior history of PVC, heart failure, VT, ventricular flutter, VF, SCD, AF, or ischemic stroke were excluded. After excluding people with missing data, 9,743,582 were included in the analysis. Figure 1 shows the flow of this study. A total of 4515 people were diagnosed with PVC, with 2334 people having PVC 1 (one outpatient record) and 2181 people having PVC 2 (two outpatient records or one inpatient record) in 2009. Baseline clinical characteristics of the cohort are summarized in Table 1. Significant differences in the baseline demographics were observed between people with and without PVC. People who developed PVC were significantly older; more likely to be female, non-drinkers, and non-smokers; and had higher prevalence of hypertension, diabetes mellitus, and dyslipidemia.

**Table 1:** Baseline demographics of patients with and without PVC.

	No PVC	All PVC (PVC 1 + PVC 2)	PVC 1	PVC 2	*p * value
	n = 9,739,067	n = 4515	n = 2334	n = 2181	(No PVC vs. all PVC)
Male sex	5,396,392 (55.41%)	2122 (47%)	1108 (47.47%)	1014 (46.49%)	< 0.001
Age ≥ 65 years	1,112,687 (11.42%)	1017 (22.52%)	475 (20.35%)	542 (24.85%)	< 0.001
*Alcohol consumption*					< 0.001
Non-drinker	5,737,180 (58.91%)	2991 (66.25%)	1543 (66.11%)	1448 (66.39%)	
Mild- to moderate-drinker	1,389,349 (14.27%)	796 (17.63%)	410 (17.57%)	386 (17.7%)	
Heavy-drinker	2,612,538 (26.83%)	728 (16.12%)	381 (16.32%)	347 (15.91%)	
*Smoking status*					< 0.001
Non-smoker	4,923,357 (50.55%)	2862 (63.39%)	1455 (62.34%)	1407 (64.51%)	
Ex-smoker	4,026,141 (41.34%)	1393 (30.85%)	751 (32.18%)	642 (29.44%)	
Current-smoker	789,569 (8.11%)	260 (5.76%)	128 (5.48%)	132 (6.05%)	
Regular physical activity	1,761,150 (18.08%)	971 (21.51%)	506 (21.68%)	465 (21.32%)	< 0.001
Diabetes mellitus	791,388 (8.13%)	484 (10.72%)	215 (9.21%)	269 (12.33%)	< 0.001
Hypertension	2,443,480 (25.09%)	2183 (48.35%)	898 (38.47%)	1285 (58.92%)	< 0.001
Dyslipidemia	1,676,816 (17.22%)	1243 (27.53%)	536 (22.96%)	707 (32.42%)	< 0.001
*BMI*					< 0.001
BMI < 0 18.5	364,514 (3.74%)	124 (2.75%)	64 (2.74%)	60 (2.75%)	
18.5 ≤ BMI < 0 23	3,831,355 (39.34%)	1656 (36.68%)	901 (38.6%)	755 (34.62%)	
23 ≤ BMI < 0 25	2,396,606 (24.61%)	1158 (25.65%)	599 (25.66%)	559 (25.63%)	
25 ≤ BMI < 0 30	2,806,442 (28.82%)	1421 (31.47%)	695 (29.78%)	726 (33.29%)	
30 ≤ BMI	340,150 (3.49%)	156 (3.46%)	75 (3.21%)	81 (3.71%)	
Chronic kidney disease	908,897 (9.33%)	603 (13.36%)	265 (11.35%)	338 (15.5%)	< 0.001
Age	46.38 ± 13.79	53.24 ± 13.33	51.79 ± 13.63	54.79 ± 12.82	< 0.001
Height	164.14 ± 9.2	162.43 ± 9.09	162.82 ± 9.14	162.01 ± 9.02	< 0.001
Weight	64.05 ± 11.67	63.3 ± 11.09	63.27 ± 11.16	63.33 ± 11.02	< 0.001
Systolic blood pressure (mmHg%)	122.22 ± 14.94	123.4 ± 15.39	122.62 ± 15.05	124.24 ± 15.7	< 0.001
Diastolic blood pressure (mmHg%)	76.25 ± 10.03	76.24 ± 10.18	76.04 ± 10.22	76.46 ± 10.13	0.972
Fasting glucose (mg/dL%)	96.93 ± 23.5	98.01 ± 21.82	97.31 ± 22.03	98.76 ± 21.57	0.002
Total cholesterol (mg/dL%)	195.31 ± 41.15	194.3 ± 36.3	195.41 ± 35.75	193.11 ± 36.86	0.098
High-density lipoprotein (mg/dL%)	56.56 ± 32.75	57.01 ± 38.75	57.76 ± 40.55	56.21 ± 36.72	0.353
eGFR (mL/min/1.73 m^2^)	82.58 ± 42.48	78.93 ± 30.86	80.08 ± 32.99	77.7 ± 28.36	< 0.001

*BMI* body mass index, *eGFR* estimated glomerular filtration rate, *PVC* premature ventricular contraction.

### Heart failure

**Figure 2:**
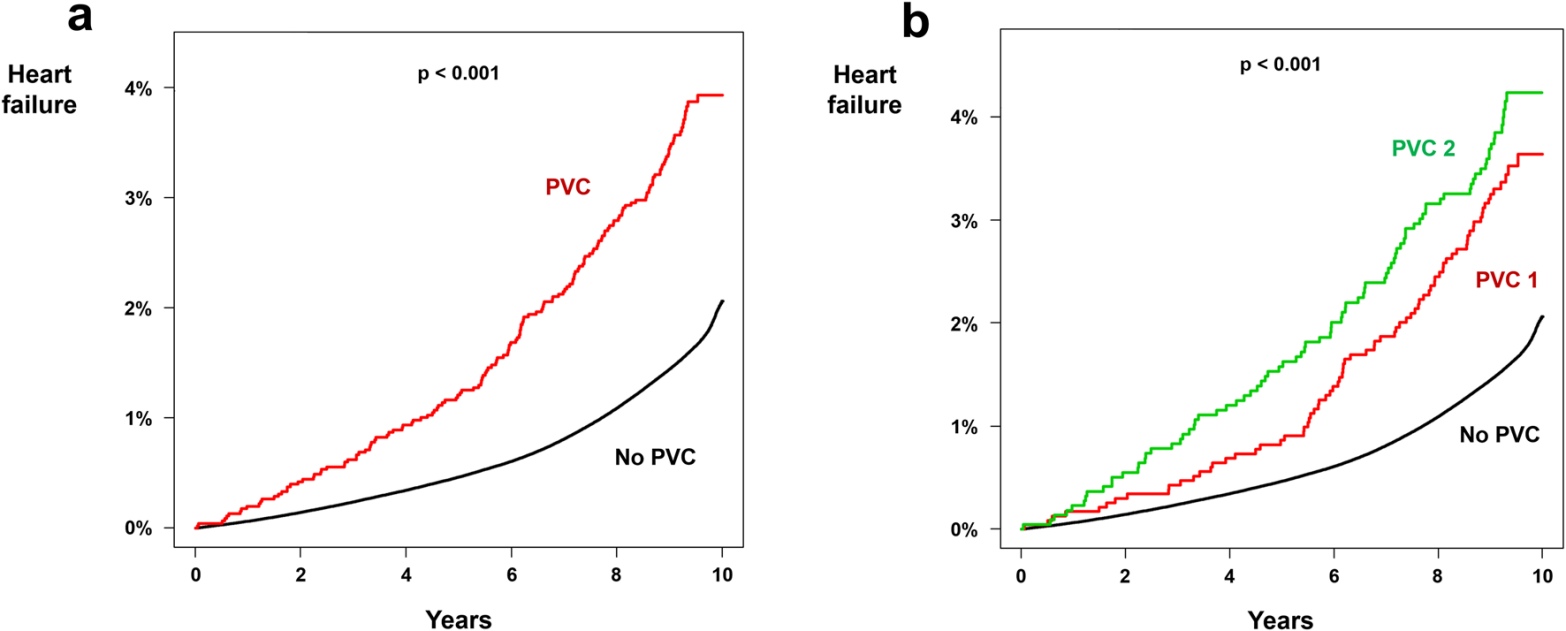
Impact of PVC on heart failure. Kaplan–Meier curve analysis showed significantly higher incidence of heart failure in people with PVC **(a)** as compared to people without PVC. People with PVC 2 had an even higher risk of developing heart failure **(b)**. PVC: premature ventricular contraction.

A total of 154,493 newly detected heart failures developed in people without PVC during the 89,450,468 person * year follow-up (incidence = 1.727). In people with PVC, 165 newly detected heart failure occurred during the 40,899 person * year follow-up (incidence = 4.034; non-adjusted HR  2.344; *p * < 0.001). Kaplan–Meier curve analysis also showed significantly higher cumulative incidence of newly detected heart failure in people with PVC (*p *< 0.001; Fig. 2a). After multivariate adjustment, presence of PVC was a significant risk factor for heart failure (HR  1.371; 95% CI 1.177–1.598; *p * < 0.001; Table 2). Both PVC 1 (HR  1.434; 95% CI 1.150–1.788; *p * < 0.001; Fig. 2b) and PVC 2 (HR  1.319; 95% CI 1.067–1.629; *p * < 0.001; Fig. 2b) had a significantly increased risk of heart failure compared with people without PVC. Subgroup analysis revealed a significant interaction between PVC and age, with people younger than 65 years having significantly higher risk of developing heart failure due to PVC (p for interaction = 0.004; Supplementary Table S2).

**Table 2:** Incidence of heart failure and ventricular arrhythmia composite in people with PVC.

	n	Event number	Follow-up duration (person * years)	Incidence	Model 1	Model 2	Model 3	Model 4
*Heart failure*								
No PVC	9,739,067	154,493	89,450,468	1.727	1 (reference)	1 (reference)	1 (reference)	1 (reference)
All PVC (PVC 1 + PVC 2)	4515	165	40,899	4.034	2.344 (2.013–2.729)	1.439 (1.235–1.676)	1.46 (1.253–1.701)	1.371 (1.177–1.598)
PVC 1	2334	79	21,278	3.713	2.150 (1.724–2.679)	1.421 (1.140–1.772)	1.459 (1.170–1.819)	1.434 (1.150–1.788)
PVC 2	2181	86	19,621	4.383	2.563 (2.077–3.163)	1.455 (1.178–1.797)	1.461 (1.183–1.805)	1.319 (1.067–1.629)
*Ventricular arrhythmia composite*								
No PVC	9,739,067	25,291	89,718,315	0.282	1 (reference)	1 (reference)	1 (reference)	1 (reference)
All PVC (PVC 1 + PVC 2)	4515	92	40,922	2.248	7.967 (6.492–9.777)	5.952 (4.852–7.303)	5.932 (4.833–7.280)	5.588 (4.553–6.859)
PVC 1	2334	28	21,337	1.312	4.665 (3.222–6.754)	3.656 (2.524–5.296)	3.659 (2.526–5.301)	3.573 (2.467–5.176)
PVC 2	2181	64	19,585	3.268	11.616 (9.092–14.839)	8.162 (6.387–10.432)	8.145 (6.373–10.41)	7.420 (5.805–9.484)

Incidence is per 1000 person * years.

Model 1 is without multivariate adjustment.

Model 2 is adjusted for age and sex.

Model 3 is adjusted for age, sex, BMI, smoking status, alcohol consumption, and physical activity.

Model 4 is adjusted for age, sex, BMI, smoking status, alcohol consumption, physical activity, hypertension, diabetes, and dyslipidemia.

*AF* atrial fibrillation, *PVC* premature ventricular contraction.

### Ventricular arrhythmia composite

**Figure 3:**
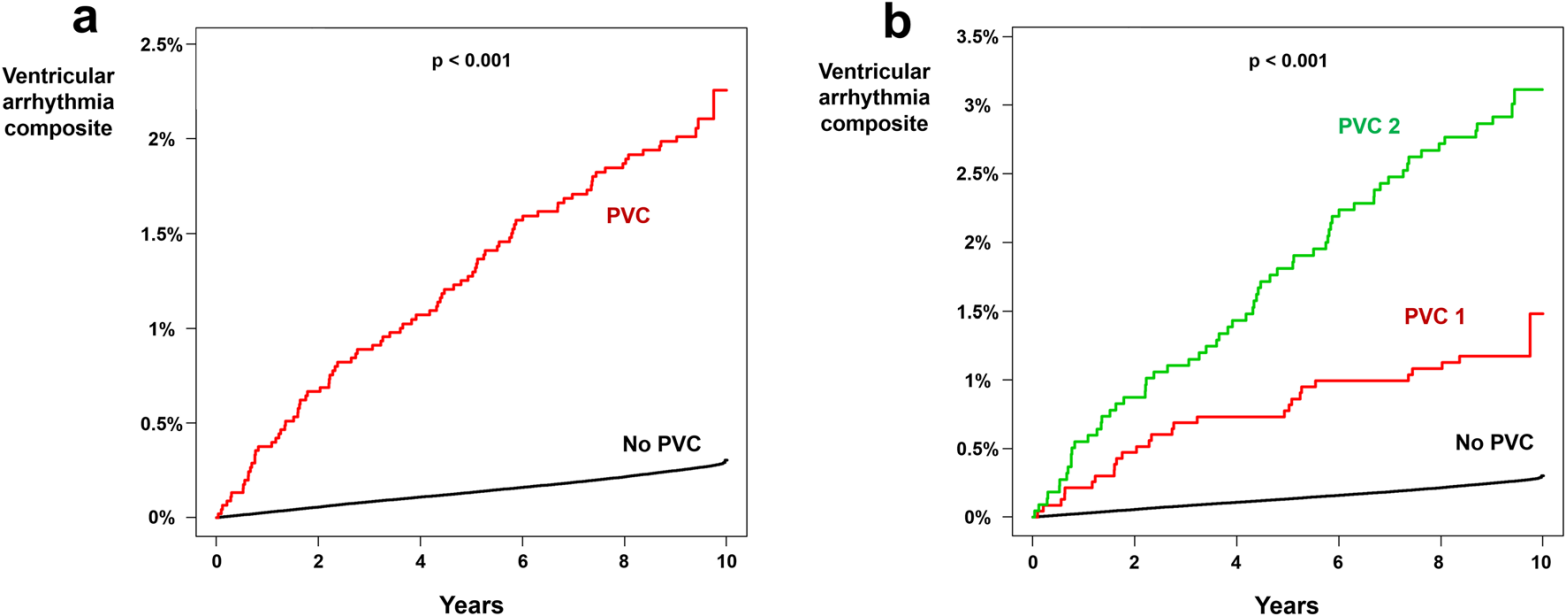
Impact of PVC on ventricular arrhythmia composite. Kaplan–Meier curve analysis showed significantly higher incidence of ventricular arrhythmia composite in people with PVC **(a)** as compared to people without PVC. People with PVC 2 had an even higher risk of developing ventricular arrhythmia composite **(b)**. PVC: premature ventricular contraction.

A total of 25,291 ventricular arrhythmia composite (VT, ventricular flutter, and VF) occurred during the 89,718,315 person * year follow-up in people without PVC (incidence = 0.282). In people with PVC, 92 ventricular arrhythmia composite occurred during the 40,922 person * year follow up (incidence = 2.248; non-adjusted HR  7.967; 95% CI 6.492–9.777; *p * < 0.001; Table 2 and Fig. 3a). After multivariate adjustment, PVC was associated with a 5.588-fold increase in the risk of ventricular arrhythmia composite (95% CI 4.553–6.859; *p * < 0.001; Table 2). Both PVC 1 (HR  3.573; 95% CI  2.467–5.176; *p * <  0.001; Table 2 and Fig. 3b) and PVC 2 (HR  7.420; 95% CI  5.805–9.484; *p * <  0.001; Table 2 and Fig. 3b) were associated with significantly increased risk of ventricular arrhythmia composite and PVC 2 was associated with greater risk of ventricular arrhythmia composite as compared to people with PVC 1. In subgroup analysis, significant interactions were observed between PVC and age: young age people at greater risk of developing ventricular arrhythmia composite for having PVC (Supplementary Table S3).

In this study, PVC group did not show any significant difference in all-cause mortality after multivariate adjustment (HR  0.939; 95% CI 0.831–1.060; Supplementary Table S4).

## Discussion

With this cohort of 9,743,582 people, we report that presence of PVC is associated with significantly higher incidence of heart failure and ventricular arrhythmia composite (VT, ventricular flutter, and VF). Chronological demonstration of the increased incidence of heart failure and ventricular arrhythmia composite based on a nationwide cohort data is the strength of our study. We performed subgroup analyses and found that age had significant interactions with PVC in developing heart failure and ventricular arrhythmia composite. People with prior diagnosis of PVC identified by ICD-10 codes will have heterogeneous duration of PVC. Therefore, we excluded people who had a prior diagnosis of PVC during 2002 to 2008 and analyzed those who were newly diagnosed with PVC in 2009. With sufficient duration of follow-up and large sample size, we were able to analyze chronological influence of newly diagnosed PVC on heart failure and lethal ventricular arrhythmias.

### Heart failure

Prior studies reported a significant improvement of left ventricular systolic function after successful suppression of PVC^8,13,14^. The diagnosis of PVC-induced cardiomyopathy in these studies are all based on inference: the diagnosis was made after improvement in left ventricular systolic function with PVC suppression^15^. In a recent analysis, improved survival was observed when PVC-induced cardiomyopathy was treated with amiodarone.

Previous observational study reported a 71% increased risk of heart failure in people with PVC with annual incidence close to 1%^16^. Another study reported that 4% among patients with PVC eventually developed left ventricular dysfunction during median 60.0 months^17^. Our results are in accordance with prior studies and demonstrated that the incidence of heart failure is significantly higher in people with PVC (HR  1.371). The presence of PVC can be a marker of preexisting cardiomyopathy. To rule out this possibility we excluded people with medical history of heart failure during the screening period. Prior diagnosis of PVC was also an exclusion criterion since these people could have had PVC for several years and might already have sub-clinical cardiomyopathy related with PVC. Therefore, we analyzed the incidence of heart failure in people who was diagnosed with new-onset PVC in 2009. We believe this selection process helped to reveal the true impact of PVC on development of heart failure.

The underlying mechanism of PVC-induced cardiomyopathy remains controversial. In theory, mechanistic features of PVC-induced cardiomyopathy and pacing-induced cardiomyopathy may be similar: a ventricular dyssynchrony^18–21^. A previous study by Leclercq et al. showed that various cardiac hemodynamics, such as cardiac output, pulmonary capillary wedge pressure, right atrial pressure, pulmonary artery pressure, and left ventricular ejection fraction, are better in AAI pacing, which preserves both normal atrioventricular and biventricular synchrony as compared to DDD or VVI pacing^21^. Intermittent dyssynchronous contraction of ventricles induced by PVC may cause adverse hemodynamic changes similar to ventricular pacing and gradually provoke cardiomyopathy and clinical heart failure.

Another important feature of PVC is post-extrasystolic potentiation a phenomenon of a transient increase in contractility following PVC. Billet et al. reported that patients with PVC-induced cardiomyopathy had higher percentage of PVCs with post-extrasystolic potentiation compared to patients with PVC but without cardiomyopathy suggesting post-extrasystolic potentiation can lead to myocardial burnout^22^. Kowlgi et al. reported that post-extrasystolic potentiation at baseline might predict future occurrence of PVC-induced cardiomyopathy as it reflects the myocardial adaptation reserve to PVCs^23^. In the other hand, the higher post-extrasystolic potentiation in patients with already developed PVC-induced cardiomyopathy indicated the lower contractile function when ectopy is not present^23^. Echocardiographic evaluation of post-extrasystolic potentiation might assist us to identify patients at risk of developing PVC-induced cardiomyopathy and to select who would benefit most from PVC suppression^23^.

Prior studies suggested that the burden of PVC is a significant risk factor for PVC-induced cardiomyopathy^3,24^. Dukes et al. reported that attributable risk of PVC for heart failure was comparable to other traditional risk factors for heart failure such as hypertension, age, and coronary artery disease especially when the burden of PVC is high^24^. Premature ventricular contraction can be suppressed by medications or radiofrequency catheter ablation, which can provide the diagnosis of PVC-induced cardiomyopathy if LV function improves or recovers^19^. Significant interaction between age and PVC was observed in our study with young people more vulnerable to adverse impact of PVC on heart failure and ventricular arrhythmias which is in accordance with prior study^25^. The underlying mechanism of such interaction is unclear but PVC in young people might represent more malignant form of PVC and therapeutic impact of aggressive suppression of PVC in young people is an area of future research.

### Ventricular arrhythmias

The incidence of ventricular arrhythmias such as VT or VF in people with PVC is not well reported. Nomura et al. reported the prevalence of PVC among 170,088 Japanese students^26^. A total of 404 students were found to have PVC in their baseline ECG and the authors obtained follow-up electrocardiogram data of 166 students. Three students among 166 developed VT during follow-up but without any statistical significance. Due to lack of follow-up data of students without PVC, they were not able to compare the incidence of VT between those with and without PVC. In people with a structurally normal heart, PVC has a benign prognosis^27,28^. However, our study revealed that people with PVC are subject to a 5.6-fold increased risk of developing lethal events such as VT, ventricular flutter, or VF. Since atrial premature contraction, especially that originating from the pulmonary veins, can induce atrial tachycardia or AF, it is not surprising that PVC can trigger VT or VF^9,29,30^. Furthermore, PVC and lethal ventricular arrhythmias might share similar substrates. Ghannam and colleagues demonstrated that the presence and amount of myocardial scarring observed in delayed enhancement cardiac magnetic resonance imaging was associated with future risk of VT^31^. Huizar and his colleagues suggested that in addition to myocardial dysfunction, electrical remodeling, autonomic dysregulation, and myocardial fibrosis by PVC can lead to increased risk of sudden cardiac death^32^. Whether PVC suppression by radiofrequency catheter ablation can reduce the incidence of sudden cardiac death is a promising area of future research since PVC suppression will not only improve heart function but can also eliminate the trigger of lethal ventricular arrhythmias^32^.

In contrast to prior report, all-cause mortality did not differ between people with and without PVC in our analysis^24^. Lack of PVC burden quantification in our study might be the cause of this disparity. In a recent post-hoc analysis of the CHF-STAT study, improved survival was observed when PVC-induced cardiomyopathy was treated with amiodarone suggesting potential association between PVC and increased risk of all-cause mortality^33^.

## Limitations

Several limitations exist in this study. First, the burden of PVC measured by Holter monitoring was not available since the K-NHIS database does not have any information about PVC burden. However, we divided people with PVC into PVC 1 and PVC 2; the diagnosis of PVC 2 was based on intensified criteria and therefore represents more severe forms of PVC. The risk of ventricular arrhythmia composite was higher in PVC 2 than PVC 1. Second, we were not able to analyze future risk of heart failure and ventricular arrhythmia composite according to the morphology or coupling interval of PVC. Dyssynchronous ventricular contraction can be affected by the origin of PVC and might impact the development of cardiomyopathy. The short coupling interval of PVC can have a higher risk of triggering VT or VF. Third, due to the nature of our study design—a retrospective analysis based on a nationwide health care database—there could be coding inaccuracies, missing data, inaccurate reporting, and selection bias, although our coding and selection strategy has been validated in multiple previous studies^11,12,34^. Fourth, the results of this study cannot be directly applied to different ethnic groups since our cohort consists exclusively of East Asians. Finally, our coding strategy for PVC needs further validation despite our prior publication using the same method to identify PVC^35^.

## Conclusions

The incidence of heart failure and ventricular arrhythmia composite is significantly increased in people with PVC. Serial outpatient follow-up of people with PVC can be helpful for early detection of heart failure and lethal ventricular arrhythmias.

## Supplementary Information

Supplementary Information.

## Data Availability

The data underlying this article are available in the article and in its online supplementary material.

## Abbreviations

AF: Atrial fibrillation
BMI: Body mass index
HR: Hazard ratio
ICD: International Classification of Disease
K-NHIS: Korean National Health Insurance Service
PVC: Premature ventricular contraction
VF: Ventricular fibrillation
VT: Ventricular tachycardia
