# Supplementary material for: Premature ventricular contraction increases the risk of heart failure and ventricular tachyarrhythmia
Source: Sci Rep. 2021 Jun 16;11:12698. doi: 10.1038/s41598-021-92088-0 (PMC8209189; doi:10.1038/s41598-021-92088-0)
Supplement: Supplementary file 1 — Supplementary Information. [file 41598_2021_92088_MOESM1_ESM.docx]

**Premature Ventricular Contraction is Associated with Increased Risk of Heart Failure and Ventricular Tachyarrhythmia: a Nationwide Population-based Study**

Yun Gi Kim,^1^ Yun Young Choi,^1^ Kyung-Do Han,^2^ Kyoung Jin Min,^1^ Ha Young Choi,^1^ Jaemin Shim,^1^ Jong-Il Choi,^1^* and Young-Hoon Kim^1^

^1^Division of Cardiology, Department of Internal Medicine, Korea University College of Medicine and Korea University Anam Hospital, Seoul, Republic of Korea.

^2^Department of Statistics and Actuarial Science, Soongsil University, Seoul, Republic of Korea.

*Address for correspondence: Jong-Il Choi, MD, PhD, MHSc

^1^Division of Cardiology, Department of Internal Medicine, Korea University College of Medicine and Korea University Anam Hospital, Seoul, Republic of Korea.

73 Goryeodae-ro, Seongbuk-gu, Seoul 02841, Republic of Korea

Tel: 82-2-920-5445

Fax: 82-2-927-1478

E-mail: [jongilchoi@korea.ac.kr](mailto:jongilchoi@korea.ac.kr)

**Brief title:** Impact of PVC on HF and VT/VF

**Total word count:** 5,236

The first two authors contributed equally to this work.

**Supplementary Table S1.** Diagnostic codes.

|  | ICD-10 codes |
| --- | --- |
| **Premature ventricular contraction** | I49.3 |
| **Heart failure** | I50 (all sub-codes) |
| **Ventricular arrhythmia composite** | I47.2, I49.0 |
| **Ventricular tachycardia** | I47.2 (all sub-codes) |
| **Ventricular fibrillation and flutter** | I49.0 |
| **Atrial fibrillation** | I48 (all sub-codes) |
| **Type 2 diabetes** | E11 – E14 (all sub-codes) |
| **Hypertension** | I10 – I13, I15 (all sub-codes) |
| **Dyslipidemia** | I78 (all sub-codes) |
| **Chronic kidney disease** | Based on creatinine checked during national health check-up |
| **Stroke** | I63, I64 (all sub-codes) |

**Supplementary Table S2.** Interaction analysis: heart failure.

| **Subgroup** | **PVC** | **n** | **Event** | **Duration**  **(person*years)** | **Incidence** | **HR** | **p for**  **interaction** |
| --- | --- | --- | --- | --- | --- | --- | --- |
| **Age < 65** | PVC (–) | 8,626,380 | 73,201 | 79,903,526 | 0.916 | 1 (reference) | 0.004 |
|  | PVC (+) | 3,498 | 76 | 32,228 | 2.358 | 1.776 (1.418 – 2.224) |  |
| **Age ≥ 65** | PVC (–) | 1,112,687 | 81,292 | 9,546,942 | 8.515 | 1 (reference) |  |
|  | PVC (+) | 1,017 | 89 | 8,671 | 10.265 | 1.156 (0.939 – 1.424) |  |
|  |  |  |  |  |  |  |  |
| **Male** | PVC (–) | 5,396,392 | 83,959 | 49,348,743 | 1.701 | 1 (reference) | 0.400 |
|  | PVC (+) | 2,122 | 78 | 18,999 | 4.105 | 1.289 (1.032 – 1.610) |  |
| **Female** | PVC (–) | 4,342,675 | 70,534 | 40,101,725 | 1.759 | 1 (reference) |  |
|  | PVC (+) | 2,393 | 87 | 21,900 | 3.973 | 1.460 (1.183 – 1.802) |  |
|  |  |  |  |  |  |  |  |
| **DM (-)** | PVC (–) | 8,947,679 | 119,082 | 82,436,389 | 1.445 | 1 (reference) | 0.014 |
|  | PVC (+) | 4,031 | 140 | 36,697 | 3.815 | 1.510 (1.280 – 1.783) |  |
| **DM (+)** | PVC (–) | 791,388 | 35,411 | 7,014,079 | 5.049 | 1 (reference) |  |
|  | PVC (+) | 484 | 25 | 4,202 | 5.950 | 0.903 (0.610 – 1.337) |  |
|  |  |  |  |  |  |  |  |
| **HTN (-)** | PVC (–) | 7,295,587 | 64,917 | 67,458,662 | 0.962 | 1 (reference) | 0.926 |
|  | PVC (+) | 2,332 | 43 | 21,462 | 2.004 | 1.374 (1.019 – 1.853) |  |
| **HTN (+)** | PVC (–) | 2,443,480 | 89,576 | 21,991,806 | 4.073 | 1 (reference) |  |
|  | PVC (+) | 2,183 | 122 | 19,437 | 6.277 | 1.368 (1.145 – 1.634) |  |
|  |  |  |  |  |  |  |  |
| **Dyslipidemia (-)** | PVC (–) | 8,062,251 | 107,847 | 74,140,326 | 1.455 | 1 (reference) | 0.027 |
|  | PVC (+) | 3,272 | 115 | 29,657 | 3.878 | 1.556 (1.296 – 1.868) |  |
| **Dyslipidemia (+)** | PVC (–) | 1,676,816 | 46,646 | 15,310,142 | 3.047 | 1 (reference) |  |
|  | PVC (+) | 1,243 | 50 | 11,242 | 4.448 | 1.077 (0.816 – 1.421) |  |
|  |  |  |  |  |  |  |  |
| **CKD (-)** | PVC (–) | 8,830,170 | 122,786 | 81,260,694 | 1.511 | 1 (reference) | 0.221 |
|  | PVC (+) | 3,912 | 116 | 35,575 | 3.261 | 1.298 (1.082 – 1.557) |  |
| **CKD (+)** | PVC (–) | 908,897 | 31,707 | 8,189,773 | 3.872 | 1 (reference) |  |
|  | PVC (+) | 603 | 49 | 5,324 | 9.203 | 1.582 (1.196 – 2.092) |  |

CKD: chronic kidney disease; DM: diabetes mellitus; HR: hazard ratio: PVC: premature ventricular contraction.

**Supplementary Table S3.** Interaction analysis: VT, ventricular flutter, or VF.

| **Subgroup** | **PVC** | **n** | **Event** | **Duration**  **(person*years)** | **Incidence** | **HR** | **p for**  **interaction** |
| --- | --- | --- | --- | --- | --- | --- | --- |
| **Age < 65** | PVC (–) | 8,626,380 | 18,178 | 80,011,964 | 0.227 | 1 (reference) | < 0.001 |
|  | PVC (+) | 3,498 | 78 | 32,078 | 2.432 | 8.042 (6.439 – 10.044) |  |
| **Age ≥ 65** | PVC (–) | 1,112,687 | 7,113 | 9,706,351 | 0.733 | 1 (reference) |  |
|  | PVC (+) | 1,017 | 14 | 8,843 | 1.583 | 2.084 (1.234 – 3.518) |  |
|  |  |  |  |  |  |  |  |
| **Male** | PVC (–) | 5,396,392 | 14,249 | 49,485,770 | 0.288 | 1 (reference) | 0.562 |
|  | PVC (+) | 2,122 | 43 | 18,999 | 2.263 | 5.098 (3.779 – 6.879) |  |
| **Female** | PVC (–) | 4,342,675 | 11,042 | 40,232,545 | 0.274 | 1 (reference) |  |
|  | PVC (+) | 2,393 | 49 | 21,923 | 2.235 | 6.059 (4.576 – 8.022) |  |
|  |  |  |  |  |  |  |  |
| **DM (-)** | PVC (–) | 8,947,679 | 21,659 | 82,630,948 | 0.262 | 1 (reference) | 0.061 |
|  | PVC (+) | 4,031 | 84 | 36,667 | 2.291 | 6.022 (4.860 – 7.463) |  |
| **DM (+)** | PVC (–) | 791,388 | 3,632 | 7,087,367 | 0.512 | 1 (reference) |  |
|  | PVC (+) | 484 | 8 | 4,255 | 1.880 | 3.148 (1.573 – 6.299) |  |
|  |  |  |  |  |  |  |  |
| **HTN (-)** | PVC (–) | 7,295,587 | 13,929 | 67,549,182 | 0.206 | 1 (reference) | 0.489 |
|  | PVC (+) | 2,332 | 34 | 21,378 | 1.590 | 6.001 (4.286 – 8.403) |  |
| **HTN (+)** | PVC (–) | 2,443,480 | 11,362 | 22,169,133 | 0.513 | 1 (reference) |  |
|  | PVC (+) | 2,183 | 58 | 19,544 | 2.968 | 5.383 (4.160 – 6.966) |  |
|  |  |  |  |  |  |  |  |
| **Dyslipidemia (-)** | PVC (–) | 8,062,251 | 18,580 | 74,313,890 | 0.250 | 1 (reference) | 0.142 |
|  | PVC (+) | 3,272 | 65 | 29,660 | 2.191 | 6.199 (4.858 – 7.909) |  |
| **Dyslipidemia (+)** | PVC (–) | 1,676,816 | 6,711 | 15,404,425 | 0.436 | 1 (reference) |  |
|  | PVC (+) | 1,243 | 27 | 11,261 | 2.398 | 4.518 (3.096 – 6.593) |  |
|  |  |  |  |  |  |  |  |
| **CKD (-)** | PVC (–) | 8,830,170 | 21,711 | 81,466,880 | 0.267 | 1 (reference) | 0.389 |
|  | PVC (+) | 3,912 | 78 | 35,549 | 2.194 | 5.805 (4.647 – 7.251) |  |
| **CKD (+)** | PVC (–) | 908,897 | 3,580 | 8,251,435 | 0.434 | 1 (reference) |  |
|  | PVC (+) | 603 | 14 | 5,373 | 2.606 | 4.675 (2.770 – 7.891) |  |

CKD: chronic kidney disease; DM: diabetes mellitus; HR: hazard ratio: PVC: premature ventricular contraction; VT: ventricular tachycardia; VF: ventricular fibrillation.

**Supplementary Table S4.** Incidence of all-cause death in people with PVC.

|  | **n** | **Event**  **number** | **Follow-up**  **duration**  **(person*years)** | **Incidence** | **Model 1** | **Model 2** | **Model 3** | **Model 4** |
| --- | --- | --- | --- | --- | --- | --- | --- | --- |
| **Heart failure** |  |  |  |  |  |  |  |  |
| No PVC | 9,739,067 | 365,147 | 89,820,879 | 4.065 | 1 (reference) | 1 (reference) | 1 (reference) | 1 (reference) |
| All PVC (PVC 1 + PVC 2) | 4,515 | 259 | 41,398 | 6.256 | 1.543 (1.366 – 1.742) | 0.920 (0.815 – 1.040) | 0.960 (0.850 – 1.084) | 0.939 (0.831 – 1.060) |
| PVC 1 | 2,334 | 111 | 21,499 | 5.163 | 1.266 (1.051 – 1.525) | 0.804 (0.667 – 0.968) | 0.847 (0.703 – 1.020) | 0.845 (0.702 – 1.018) |
| PVC 2 | 2,181 | 148 | 19,899 | 7.438 | 1.825 (1.553 – 2.144) | 1.032 (0.878 – 1.213) | 1.066 (0.907 – 1.252) | 1.023 (0.871 – 1.202) |

Incidence is per 1,000 person*years.

Model 1 is without multivariate adjustment.

Model 2 is adjusted for age and sex.

Model 3 is adjusted for age, sex, BMI, smoking status, alcohol consumption, and physical activity.

Model 4 is adjusted for age, sex, BMI, smoking status, alcohol consumption, physical activity, hypertension, diabetes, and dyslipidemia.

AF: atrial fibrillation; PVC: premature ventricular contraction.
